# Draft Genome Sequences of Seven Vibrio cholerae Isolates from Adult Patients in Qatar

**DOI:** 10.1128/MRA.01489-20

**Published:** 2021-03-04

**Authors:** Ameena Al Malki, Kyle D. Brumfield, Clement K. M. Tsui, Anjana Anand, Shah M. Rashed, Emad Ibrahim, Hamad Al Shamari, Anwar Huq, Rita R. Colwell, Rashmi Fotedar

**Affiliations:** a Department of Genetic Engineering, Biotechnology Centre, Doha, Qatar; b Maryland Pathogen Research Institute, University of Maryland, College Park, Maryland, USA; c University of Maryland Institute for Advance Computer Studies, University of Maryland, College Park, Maryland, USA; d Department of Pathology, Sidra Medicine, Doha, Qatar; e Department of Pathology and Laboratory Medicine, Weill Cornell Medicine–Qatar, Doha, Qatar; f Division of Infectious Diseases, Faculty of Medicine, University of British Columbia, Vancouver, BC, Canada; g Department of Microbiology, Hamad Medical Corporation, Doha, Qatar; h Qatar University, Biomedical Research Centre, Doha, Qatar; Loyola University Chicago

## Abstract

We report the draft genome sequences of seven Vibrio cholerae isolates from patients. Four isolates were profiled as multilocus sequence type 69, serogroup O1, a subset of seventh-pandemic El Tor clonal isolates. Presented here are genome assemblies and evidence for major pathogenicity islands, virulence factors, and antimicrobial resistance genes.

## ANNOUNCEMENT

Cholera is an acute diarrheal disease and is transmitted via untreated water carrying the etiological agent Vibrio cholerae. Serogroups O1 and O139 are the causative agents of the ongoing pandemic, the seventh, and sporadic outbreaks globally (1). V. cholerae O1 isolates can be classified as the classical or El Tor biotype based on genotypic and phenotypic characteristics (2). Since the 19th century, seven cholera pandemics have been recorded, and V. cholerae O1 El Tor is the most common serogroup (1, 3). Although cholera is endemic across Africa and Asia, the disease causes a serious public health burden in many places. However, V. cholerae has not been reported in Qatar.

Here, we report draft genome assemblies of seven V. cholerae strains from adults with cholera-like symptoms at Hamad Medical Corporation, Doha, Qatar. Strain H08 was isolated from blood, and six isolates were from stool. Briefly, swabs were inoculated onto mSuperCARBA solid medium (CHROMagar, France) and incubated under aerobic conditions at 35 ± 2°C for 18 to 24 h, minimizing exposure to light. After incubation, blue colonies were confirmed using the matrix-assisted laser desorption ionization–time of flight (MALDI-TOF) Biotyper system (Bruker, MA). Antimicrobial susceptibility was determined using the Phoenix system (Becton, Dickinson, NJ). MICs for antibiotics were determined according to the CLSI breakpoints for *Vibrio* spp. (4). All sequence type 69 (ST69) isolates showed some level of resistance to the commonly employed antibiotics (4). *Vibrio* cultures were maintained in Difco LB broth (Fisher Scientific, Hampton, NH) with aeration at 37°C.

Genomic DNA was extracted using a ZymoBIOMICS DNA miniprep kit (Zymo Research, CA), and the concentrations were determined using a Qubit 4.0 fluorometer (Thermo Fisher Scientific, Waltham, MA). DNA libraries were constructed using the IonXpress Plus fragment library kit; they were enriched and barcoded using the IonXpress barcode adapter kit (Thermo Fisher, MA). PCR products were purified using SPRIselect reagent (Beckman Coulter, Indianapolis, IN). Sequencing was performed on an Ion S5 XL semiconductor sequencer (Ion Torrent; Thermo Fisher Scientific) to generate 200-bp sequence reads. Adapter sequences were removed, and low-quality bases were trimmed using Trim Galore v.0.6.5 (http://www.bioinformatics.babraham.ac.uk/projects/trim_galore/). Read quality was confirmed using FastQC v.0.11.9 (https://github.com/s-andrews/FastQC), and the reads were assembled using SPAdes v.3.9.0 (5) with the options “–careful,” to reduce the number of misassembles, and “–cov-cutoff auto,” to remove misassembled low-coverage contigs. Small contigs (<500 bp) were discarded. Assembly statistics were assessed using QUAST v.5.0.2 (6). Multilocus sequence types (MLST) and antimicrobial resistance genes were predicted using the mlst (https://github.com/tseemann/mlst) and ResFinder v.3.2 databases (7) through abricate v.0.9.8 (https://github.com/tseemann/abricate) based on ≥70% coverage and ≥90% sequence identity. Any unknown MLST sequences that did not match the existing alleles were submitted to pubMLST (https://pubmlst.org/vcholerae/). Virulence genes, pathogenicity islands (8), and O1 and O139 antigen-encoding genes were typed using CholeraeFinder v.1.0 (https://cge.cbs.dtu.dk/services/CholeraeFinder-1.0/). The genomes were annotated using the NCBI Prokaryotic Genome Annotation Pipeline v.4.11 (9). Default parameters were used for all software unless otherwise specified.

Genome statistics and information for the seven isolates are summarized in Table 1. Four isolates (H03, H06A, H09, and H12) were identified as ST69, possessing genomic islands (GI) VSP-1, VSP-2, VPI-1, and VPI-2, as well as toxin/virulence genes, including *toxR*, *tcpA*, *rtxA*, *hlyA*, and *ctxA*, characteristics of seventh-pandemic V. cholerae O1 El Tor (1). All of the O1 El Tor isolates also carried *sul2*, *tet*(59), *dfrA1*, *catB9*, *aph(3″)-Ib*, and *aph(6)-Id*, which code for resistance to the antibiotics sulfamethoxazole, tetracycline, trimethoprim, chloramphenicol, and streptomycin. Isolates H01, H08, and H10, however, were profiled as non-O1/non-O139. These isolates provide baseline information on the diversity of V. cholerae in Qatar.

**TABLE 1:** Summary of genome statistics and genetic mechanism of antibiotic resistance

Isolate no./GenBank accession no.	No. of reads	Genome size (bp)	*N*_50_ (bp)	GC content (%)	Avg coverage (×)	No. of contigs	No. of CDS^*a*^	Multilocus sequence type	Predicted antimicrobial resistance genes (ResFinder)
H01/JACWAP000000000	4,174,356	4,034,608	194,613	47.53	176	80	3,629	1287	*tet*(34)
H03/JACWAQ000000000	3,715,840	4,040,646	122,900	47.47	129	99	3,633	69	*tet*(34), *sul2*, *tet*(59), *dfrA1*, *catB9*, *aph(3″)-Ib*, *aph(6)-Id*
H06A/JACWAR000000000	7,778,339	4,041,525	170,527	47.47	348	75	3,629	69	*tet*(34), *sul2*, *tet*(59), *dfrA1*, *catB9*, *aph(3″)-Ib*, *aph(6)-Id*
H08/JACWAS000000000	15,621,973	3,935,466	529,619	47.53	700	60	3,536	1296	*tet*(34)
H09/JACWAT000000000	5,559,609	4,042,871	141,878	47.47	192	102	3,646	69	*tet*(34), *sul2*, *tet*(59), *dfrA1*, *catB9*, *aph(3*″*)-Ib*, *aph(6)-Id*
H10/JACWAU000000000	7,684,224	4,133,920	170,440	47.46	252	87	3,695	1301	*tet*(34)
H12/JACWAV000000000	18,701,382	4,027,045	180,171	47.51	900	84	3,620	69	*tet*(34), *sul2*, *tet*(59), *dfrA1*, *catB9*, *aph(3*″*)-Ib*, *aph(6)-Id*

a CDS, coding DNA sequences.

### Data availability.

The whole-genome shotgun data from this study have been deposited in the DDBJ/ENA/GenBank repositories under SRA number PRJNA656914. The genome assembly versions described in this paper are the first versions.
